# High-resolution computed tomographic analysis of tooth replacement pattern of the basal neoceratopsian *Liaoceratops yanzigouensis* informs ceratopsian dental evolution

**DOI:** 10.1038/s41598-018-24283-5

**Published:** 2018-04-12

**Authors:** Yiming He, Peter J Makovicky, Xing Xu, Hailu You

**Affiliations:** 1 School of Earth Sciences and Engineering, Nanjing University, Nanjing, Jiangsu 210046 China; 2 Department of Geology, The Field Museum, Chicago, llinois 60640 United States of America; 3 Key Laboratory of Vertebrate Evolution and Human Origins, Institute of Vertebrate Palaeontology and Paleoanthropology, Chinese Academy of Sciences, Beijing, 100046 China

## Abstract

The dental morphology and tooth replacement pattern of *Liaoceratops yanzigouensis*, the earliest known neoceratopsian, are important for our understanding of the evolution of the ceratopsian dental system. Here we describe the dental morphology and tooth replacement of *Liaoceratops yanzigouensis* based on high-resolution computed tomographic (CT) scan data of three specimens including the holotype, the first study for basal ceratopsian. The three-dimensional reconstructions reveal some important new information, including: three teeth in the premaxilla in one side, two more teeth in the dentary than in the maxilla, incipiently developed mesial grooves on some crowns, two generations of replacement teeth within some tooth families; and most functional teeth were under heavy resorption by the replacement process, but still remained functional. Comparisons of tooth pair positions from opposite sides in the four jaw quadrants of three specimens revealed a degree of bilateral symmetry in replacement pattern. Reconstruction of Zahnreihen yields an avergae z-spacing of 2.58 with simultaneous front-to-back tooth replacement. Our study presents the earliest evidence of derived neoceratopsian traits of the complex dental batteries in ceratopsids. Most significantly, our models reveal the tracts of partially resorbed functional teeth which appears to track the growth of the jaws, traits previously undocumented in Ceratopsia.

## Introduction

The Ceratopsia is one of the most diverse dinosaur clades in the Cretaceous and played a significant role in the terrestrial ecosystems of Asia and western North America^1–4^. Most ceratopsians belong to the Neoceratopsia, which includes the large bodied Late Cretaceous Ceratopsidae, a defining feature of which is the unique dental battery. Ceratopsid jaws contain large numbers of teeth, which are mesiodistally compressed for close packing in dental batteries in which files of teeth interlock both vertically and horizontally^5,6^. The presence of such a tooth battery is considered key to their radiation in the Late Cretaceous of North America and Asia. In contrast, basal neoceratopsians lack dental batteries. Tooth replacement rates in basal neoceratopsians and ceratopsids are obviously different; however, previous studies have been restricted largely to the morphological description of functional teeth and some erupted replacement teeth that were exposed in lingual view.

*Liaoceratops yanzigouensis* from the Lower Cretaceous Yixian Formation in western Liaoning Province, northeastern China is the most basal member of Noeceratopsia^7^, and exhibits both features of derived neoceratopsians and its sister group *Psittacosaurus*, and thus plays an important role in our understanding of the origin and early evolution of neoceratopsians, including our understanding of the evolution of the ceratopsian dental system.

The dentition and tooth replacement pattern of a few basal ceratopsians was described briefly by Edmund^8^, and Tanoue *et al*.^4,9^ added new information on the two species *Auroraceratops* and *Liaoceratops*. However, the restrictions imposed by destructively sampling tooth-bearing bones hampered the amount of information that could be gleaned from traditional methods. We used computed tomography (CT), which is a nondestructive technique, to study the *in situ* dentition of the tooth-bearing bones of *Liaoceratops*, and this is to our knowledge the first study of the internal structure of the dention in a basal ceratopsian. By employing three-dimensional (3D) visualization techniques, CT scan data allow for an accurate, digital restoration and reconstruction of the tooth replacement pattern in this taxon.

### Tooth replacement function and Zahnreihen-concept

Tooth replacement is a continuous process occurring throughout the life of reptiles^8^. Previous work stretching back almost 100 years provides data on pre- and post-hatching crocodiles^10^, bony fish^11^ and tuataras^12^. However, Edmund^8,13–15^ was the first to study the replacement patterns of reptiles systematically^16^. Edmund^8^ found that tooth replacement takes place in cycles and produces wave-like patterns along the jaw^17^. Edmund^8,13^ named these pattern replacement waves, in which every second tooth is part of a single replacement wave and initiation of new teeth takes place from anterior to posterior. Woerdeman^18^ was the first person to propose that teeth arise and replace in waves and coined the term ‘Zahnreihen’ to describe this pattern^8,19^. Zahnreihen have been defined as groups of teeth that are at a similar stage of development, which lie adjacent to each other but belong to different tooth families^19^.

From the reconstruction of Zahnreihen, a Z-spacing can be retrieved^14,20–24^. The Z-spacing represents the distance between the cycles of tooth replacement along the jaws^25^. It is measured as the distance of a tooth in replacement wave to the posterior following Zahnreihen. Z-spacing allows the developing dentition to be characterized quantitatively by providing information about replacement waves and progression of tooth exchange^22,23,26–31^.

Previous tooth replacement data in dinosaurs come principally from theropods^8^, and sauropodomorphs^3,4,9,22,25,30,32–40^. The tooth replacement pattern is inferred from the degree of wear plus root resorption and the relative height of the root^30,34^. To quantify the replacement stages independently of the varying height of the teeth in a specimen, we use the replacement index as Fastnacht^16^ did in studying *Coloborhynchus robustus*. A full-grown tooth without replacement tooth was assigned the value 1.0. If a replacement tooth was present, its height was set as percentage of the height of the corresponding functional tooth. This value divided by 100 yielded the replacement index. The Z-spacing was measured as the distance of a replacement index to the posterior following Zahnreihen. A spacing of 2.0 represents the ‘ideal’ or ‘perfect’ alternation^16,22^. Most reptiles show a spacing between Zahnreihen (Z-spacing) of between 1.56 and 2.80 tooth positions^20^, but this value can change considerably during ontogeny^16^.

## Material

The skull and dentary material of *Liaoceratops yanzigouensis* comes from the famous Yixian formation of Liaoning, China. The specimens used in this study are IVPP V12738 (Institute of Vertebrate Paleontology and Paleoanthropology, Chinese Academy of Sciences, Beijing, China), IVPP V12633 and CAGS-IG-VD-002^7,10^ (Chinese Academy of Geological Sciences, Institute of Geology, Beijing, China). The holotype (IVPP V12738) is an adult skull and the other two are juvenile skulls, including CAGS-IG-VD-002, which is the smallest *Liaoceratops* specimen yet described^9^. All specimens in this paper compose both skull and mandibles, but the left mandible in CAGS-IG-VD-002 was not included in the CT scanning.

### Premaxilla

**Figure 1:**
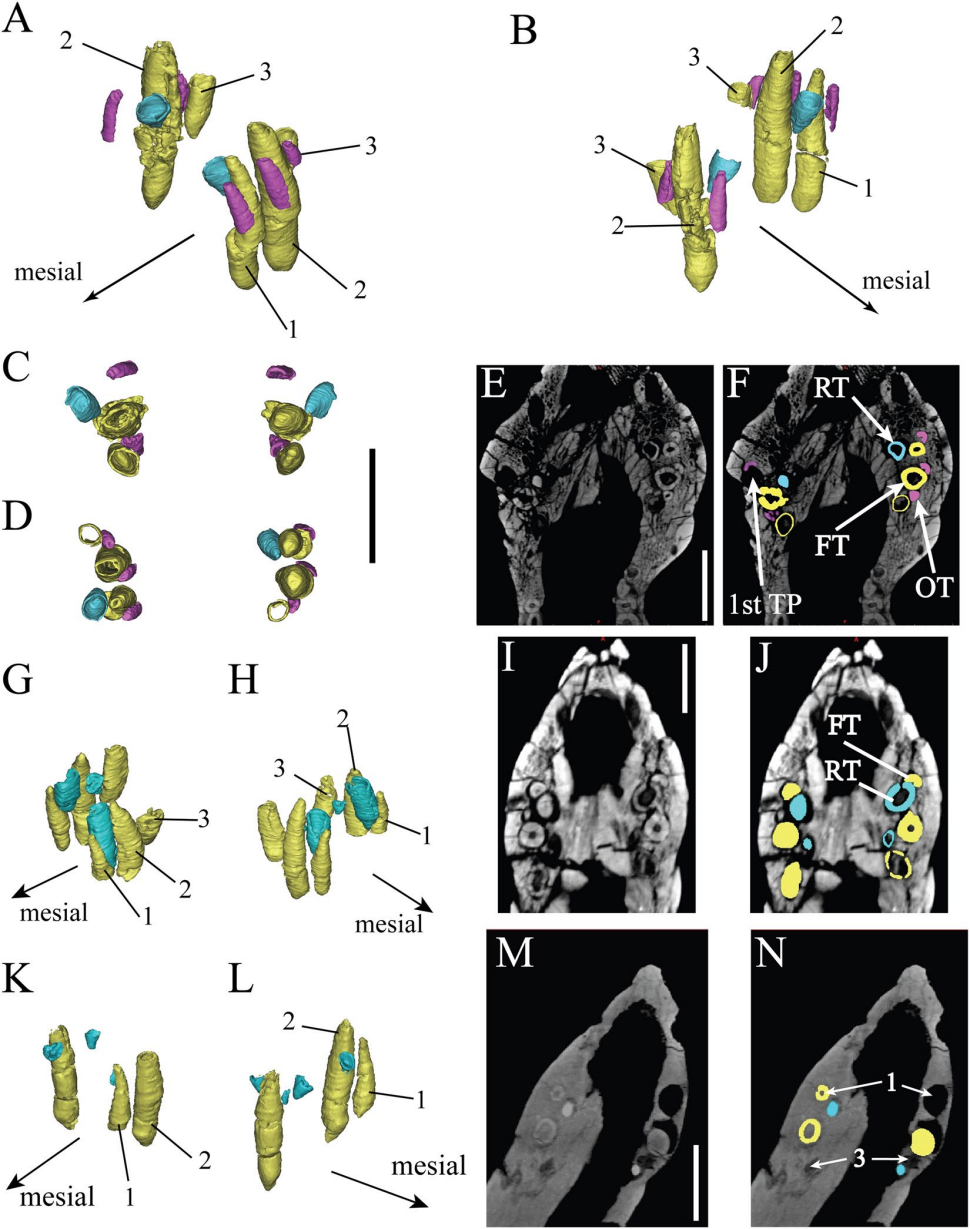
Liaoceratops yanzigouensis premaxillary teeth of IVPP V12738 (**A**–**F**), IVPP V12633 (**G**–**J**) and CAGS-IG-VD-002 (**M**–**R**). Elements in the CT reconstructions are color-coded as follows: right and left functional premaxillary teeth, yellow; right and left replacement premaxillary teeth, cyan; right and left remnants of mostly resorbed functional premaxillary teeth, magenta. Reconstructed and extracted premaxillary teeth composite in left (**A**) and right (**B**) mesial-labial view; (**C**) reconstructed and extracted left premaxillary teeth composite in dorsal (left) and ventral (right) view; (**D**) reconstructed and extracted right premaxillary teeth composite in dorsal (left) and ventral (right) view. (**E** and **F**) longitudinal CT cross sections through the premaxilla of IVPP V12738. Numbers indicate tooth positions from mesial to distal. Scale bars equal 10 mm (**C**–**F**). Reconstructed and extracted premaxillary teeth composite of IVPP V12633 in left (**G**), right (**H**) mesial-labial view; (**I**,**J)** longitudinal CT cross sections through the premaxilla of IVPP V12633. (**K**–**N**) reconstructed and extracted premaxillary teeth composite of CAGS-IG-VD-002 in left (**K**) and right (**L**) mesial-labial view; Longitudinal CT cross sections through the premaxilla of CAGS-IG-VD-002 (**M**,**N)**. Numbers indicate tooth positions from mesial to distal. Scale bars equal 5 mm (**I**,**J**,**M**,**N**).

Two premaxillary teeth are preserved on both sides in the holotype. The original first right functional premaxillary tooth (FT) has been lost and left an empty socket (Fig. 1A–F). The third socket on the left side was considered an artefact^9^, but in fact only the crown is lost and the root is visible in CT scans (Fig. 1A,B,E,F). In the two juvenile specimens (IVPP V12633 and CAGS-IG-VD-002), each premaxilla also contains three teeth^41^. In IVPP V12633, the crown of the left third functional tooth was lost (Fig. 1G–J). In CAGS-IG-VD-002, only three functional teeth are still present, two on the left and one on the right (Fig. 1K–N). The CT sections reveal no internal interalveolar septa within the premaxilla, unlike what is observed in most sauropods^25^.

The digital reconstruction shows that tooth 1 and tooth 2 on the left side of the holotype have similar heights (Table 1). The third functional tooth on the right side is quite short and reaches only one-third the height of the second tooth. All well- developed premaxillary teeth are slightly labiolingually compressed with an oval outline in cross-section and a narrow root tip in dorsal view (Fig. 1C,D).

**Table 1:** Premaxillary teeth in Liaoceratops yanzigouensis.

Tooth position	IVPP V12738		IVPP V12633		CAGS-IG-VD-002	
(TP)	left	right	left	right	left	right
FPm 1	14.89	n.p.	5.21	8.33	5.85	n.p.
FPm 2	15.69	17.04	8.78	12.15	9.05	9.31
FPm 3	2.24↑	4.76	6.14↑	6.34↑	n.p.	n.p.
RPm 1	n.d.	3.86	16.57	4.93	1.23	1.54
RPm 2	n.d.	n.d.	1.37	2.32	n.d.	n.d.
RPm 3	n.d.	n.d.	n.d.	n.d.	n.d.	1.20

n.p. = not preserved.

n.d. = not developed.

↑ = the true size should be higher as the crowns were lost.

All measurement in mm.

**Figure 2:**
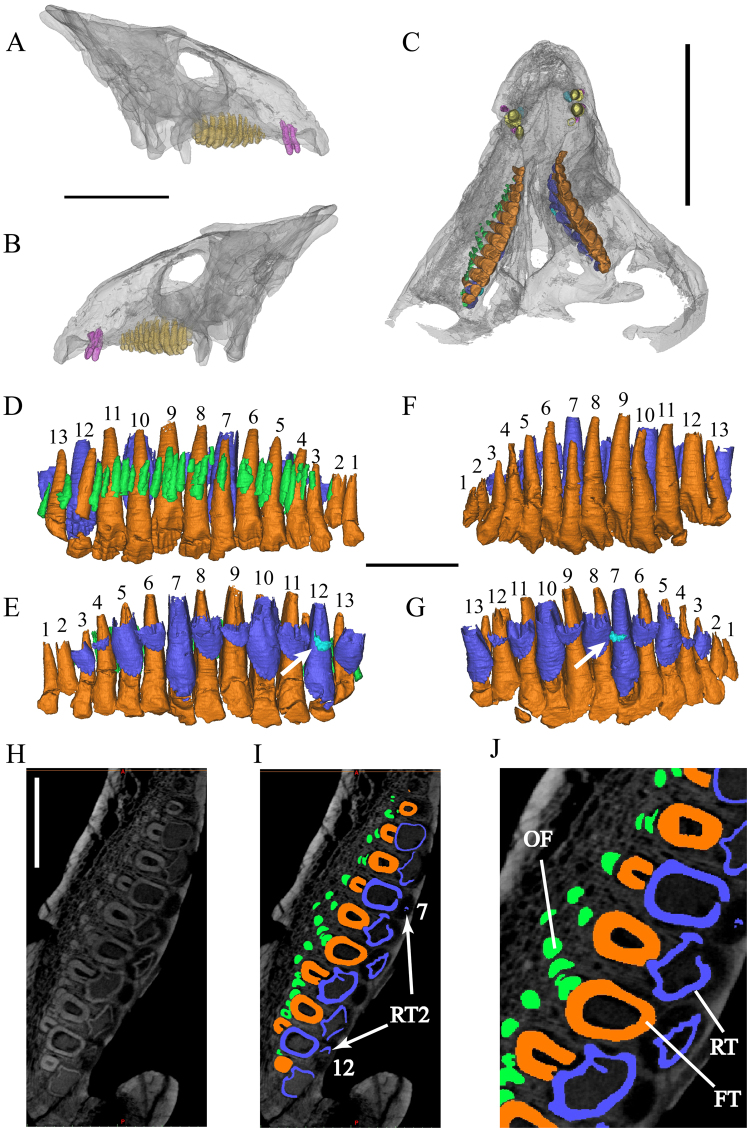
*Liaoceratops yanzigouensis*, IVPP V12738 (holotype) maxillary teeth. Elements in the CT reconstructions are colour-coded as follows: functional teeth (**A** and **B**), yellow; functional teeth (**C**–**J**), orange; 1^st^ generation replacement teeth, blue; 2nd generation replacement teeth, turquoise; right old resorbed functional teeth, green. (**A**,**B**) Transparent reconstruction of the skull of IVPP V12738 from 450 kV CT data in lateral view; (**C**) transparent reconstruction of the maxillary teeth portion of IVPP V12738 from 225 kV CT data in ventral view; (**D**) (right), (**F**) (left), reconstructed and extracted maxillary teeth composite in labial view; (**E**) (right), (**G**) (left), reconstructed and extracted maxillary teeth composite in lingual view; (**H**–**J**) longitudinal CT cross sections through the right maxilla, (**J**) zoomed photograph of **I**. Numbers indicate tooth positions from mesial to distal. Scale bars equal 50 mm (**A**–**C**) and 10 mm (**D**–**J**).

Two replacement premaxillary teeth (RT) can be seen in CT sections in the holotype. They are positioned lingual to their corresponding functional teeth but have not had not caused any root resorption at this stage. The replacement tooth of the right first alveolus is slightly inclined so that its crown is adjacent to the lingual wall of the functional tooth root (Fig. 1C,D). The apices of replacement teeth are more acuminate than those of the functional teeth. In the premaxilla of IVPP V12633, four replacement teeth are present. The replacement tooth of left functional tooth 1 is nearly three times taller than the corresponding functional crown, which is heavily resorbed and about to be shed. Four replacement teeth are present in the specimen CAGS-IG-VD-002 (Fig. 2K–N), all of them are newly erupted, cone-shaped crowns with and accompanied by their predecessors.

There are several remnants of resorbed functional premaxillary teeth (old functional teeth, OF) visible in CT sections of the holotype (Fig. 1A–F). These remains have a semi-circular cross section and are situated adjacent to the mesiolabial side of the functional premaxillary teeth. No remains of resorbed functional premaxillary teeth are observed in either of the two juvenile specimens (Fig. 1G–N).

### Maxilla

**Figure 3:**
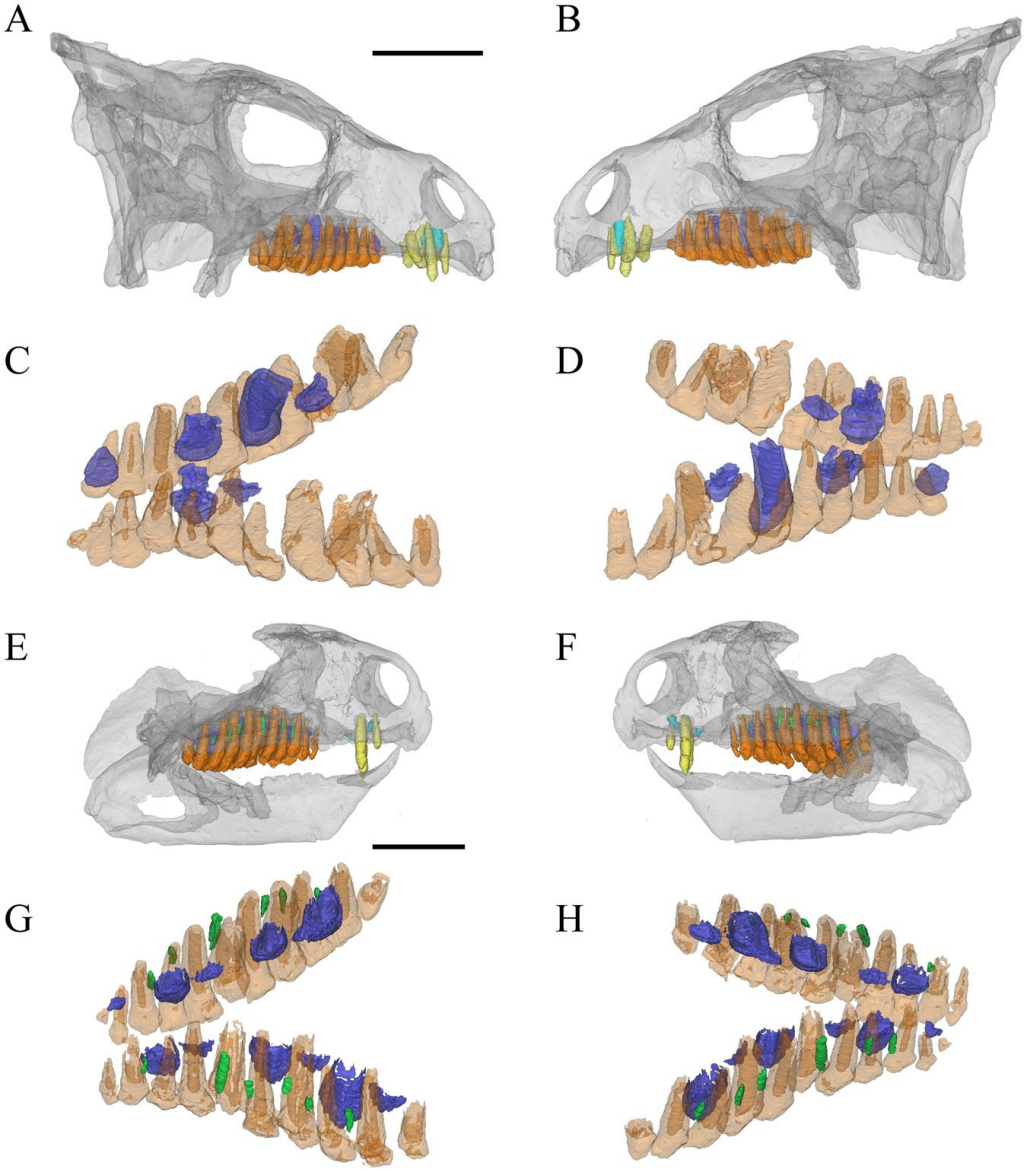
*Liaoceratops yanzigouensis*, IVPP V12633 and CAGS-IG-VD-002 maxillary teeth. Elements in the CT reconstructions are colour-coded as follows: functional premaxillary teeth, yellow; replacement premaxillary teeth, cyan; remnants of mostly resorbed functional premaxillary teeth, magenta; functional teeth, orange; replacement teeth, blue; resorbed functional teeth, green. (**A**,**B**) transparent reconstruction of the skull of IVPP V12633 in lateral view; (**C**,**D**) reconstructed and extracted maxillary teeth composite in dorsal-labial view; (**E**,**F**) transparent reconstruction of the skull of CAGS-IG-VD-002 in lateral view; (**G**,**H**) reconstructed and extracted maxillary teeth composite of CAGS-IG-VD-002 in dorsal-labial view. Scale bars equal 20 mm.

Both the left and right maxilla of the holotype (Fig. 2) contain 13 functional teeth (Fig. 2D–G), whereas both juvenile specimens bear 10 functional teeth in each maxilla^9^ (Fig. 3). Replacement teeth are present at almost every functional tooth position except for the first and second tooth positions in the holotype. There are 6 replacement teeth present on one side in IVPP V12633 and in CAGS-IG-VD-002.

The preservation of maxillary teeth in the holotype is quite complete though taphonomic distortion makes the tooth row unnaturally curved. In the holotype, the first and second teeth are relatively small. The first and second teeth on the left are only visible in CT section (Fig. 2D,F). The remaining teeth increase in size to a maximum in the eighth and ninth teeth, then decrease in size, with the last tooth being similar in size to the third tooth.

**Figure 4:**
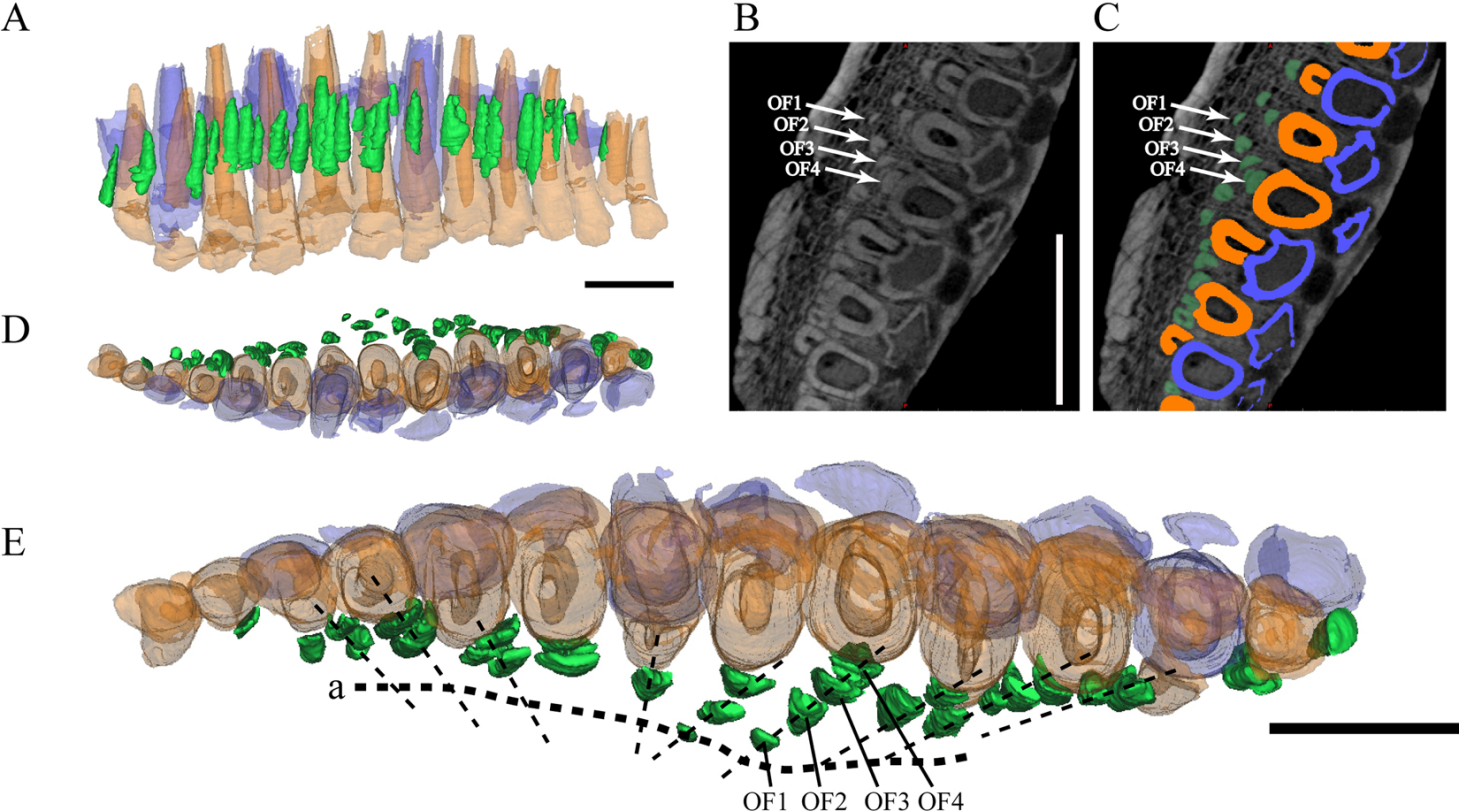
The remains of resorbed functional teeth in *Liaoceratops yanzigouensis*. Right maxilla of IVPP V12738. Elements in the CT reconstructions are colour-coded as follows: functional teeth, orange; replacement teeth, blue; old resorbed functional teeth, green. Transparent reconstructed and extracted right maxillary teeth composite in labial view (**A**), dorsal view (**D**) and ventral view (**E**). (**B**,**C**) longitudinal CT cross sections through the right maxilla. The dotted line (**E**) represent the possible growth track of the functional teeth and their predecessors. Scale bars equal 10 mm.

Our 3D reconstruction shows the complete morphology of every functional tooth (Figs 2–4). All teeth have a subcircular cross section and are widest mesimat their crown bases and taper apically to form the elongate roots. The crown cross sections reveal a pulp cavity surrounded by a thick layer of dentine. The cross sections become mesiodistally compressed and have a relatively small apical opening. The roots display incipiently developed grooves along the mesial and distal surfaces, which are seen in more derived neoceratopsians^25^. In the holotype specimen, strong root resorption is seen in left functional teeth 5, 7, 10, and 12 in lingual view by 3D reconstruction (Fig. 2E,G). Their dentine has been resorbed by the erupting replacement teeth from the lingual side, which breaches the pulp cavity. Their bases have been hollowed, out but the crowns are still functional during most of the resorption process. The right functional tooth 12 of the holotype has been bisected by the replacement tooth such that the crown tip is widely separated from the remnant of its root (Fig. 2E).

CT data reveal all replacement teeth inside the maxilla of the holotype specimen (Figs 2–4). The pulp cavity is surrounded by a thin layer of dentine, which becomes even thinner towards the base of the root, especially in some mature teeth in the process of eruption. A second replacement tooth occurs medial to left functional tooth 7 and right functional tooth 12 in the holotype (Fig. 2J). These two replacement teeth are not well developed and consist only of the crowns with no roots.

Remnants of partially resorbed functional teeth have only recently been reported in studies on the tooth implantation and replacement in dinosaurs, including small amounts of dentine buried in interdental bone in dentary of the theropod *Coelophysis*^42^, and a partially resorbed tooth at the tip of the lower jaw in a hadrosaurid specimen^43^. The holotype specimen of *Liaoceratops yanzigouensis* is unusual in that it preserves several generations of partially resorbed functional teeth in both maxillae. They can be seen clearly in the longitudinal CT cross sections through right maxilla of IVPP V12738 (Fig. 2C,E,H–J), and CAGS-IG-VD-002. Most of these resorbed teeth have a semicircular shape in longitudinal view and exhibit a greater density than the spongy bone surrounding the roots. Some remnants can clearly be identified as partial roots of resorbed teeth, because they preserve a pulp cavity surrounded by dentine (Fig. 4). The pulp cavity is small, with a narrower diameter than either the erupted functional tooth or adjacent, younger generations of resorbed tooth remnants. Resorbed functional teeth in the left maxilla and dentary of IVPP V12738 together with those in the maxilla of IVPP V12633 were not rendered as 3D reconstructions, because for they are difficult to discern in the scan data, possiby due to taphonomic distortion. The remants of resorbed teeth are most numerous in the maxilla of holotype IVPP V12738 among the three scanned specimens. The remnants are positioned labiodistal to functional teeth 2 through 6, others are positioned mesiolabial to functional teeth 8 to 13 except one, which lies just labial to tooth 7 (Figs 2H–J, 4B–E). There are between one to four generations of resorbed tooth remnants along the maxillary tooth row. The greatest number is found adjacent to tooth position 9. Four remnants are also associated with tooth position 11, with three remnants associated with tooth positions 5 and 8. The other tooth positions are associated with one (positions 1, 7, 13) or two (positions 3, 4, 6, 10, 12) remnants (Fig. 4E). In contrast, there is at most one generation of resorbed tooth remnants for any maxillary tooth position in each of the two juvenile specimens (Fig. 3).

The number and orientation of the resorbed tooth fragments (Fig. 4E) appear to track the growth of the maxilla and the progressive addition of tooth positions at the caudal, and especially, the rostral end of the tooth row. The tooth positions with the largest number of resorbed root remnants (9, 11) likely represent tooth positions that were present at or near the time of hatching, whereas tooth positions at the rostral and caudal ends of the tooth row with fewer generations of resorbed replacement tooth remnants were added later in ontogeny. The order in which tooth positions are added according as indicated by the presence and number of resorbed tooth generations is broadly similar to observations published for *Alligator*^44–46^. We note, however, that considerable variance exists along the tooth row, such that some ontogentically old tooth positions such as tooth position 10 in the holotype may not have a large number of resorbed remnants associated with them (Fig. 4E). Further study on the histology of teeth may further illuminate the rate of tooth replacement and tooth position addition in the ontogeny of *Liaoceratops*.

### Dentary

Each dentary of IVPP V12738 holds 15 tooth positions. IVPP V12633 and CAGS-IG-VD-002 both have 12 teeth in each dentary. There are thus two more tooth positions in the dentary than the maxilla in each specimen (Tables 2–7). The left mandible of CAGS-IG-VD-002 is disarticulated from the rest of the skull so it was not CT scanned. The dentary dentitions and their replacement patterns are similar to those of the maxillary teeth^4^. The mandibles of the holotype are strongly taphonomically distorted and we are therefore unable to identify any remnants of resorbed functional teeth in the CT-sections.

**Table 2:** Tooth height and the R/F ratio in the upper jaws of IVPP V12738.

Number of alveolus	Total height of functional tooth (F)		Total height of replacement tooth (R)		R/F ratio	
	left	right	left	right	left	right
1	5.90	9.42	n.d.	n.d.	n.d.	n.d.
2	6.79	7.21	n.d.	n.d.	n.d.	n.d.
3	12.22	11.43	4.39	4.51	0.36	0.39
4	13.48	13.34	1.37	1.55	0.1	0.12
5	16.65	15.17	8.65	8.42	0.52	0.56
6	17.56	15.94	3.83	3.64	0.22	0.23
7	16.08	15.92	13.81/0.95	13.16	0.86/0.06	0.83
8	18.73	17.60	6.07	6.34	0.32	0.36
9	18.41	17.11	2.92	3.06	0.16	0.18
10	17.51	17.60	10.65	10.44	0.61	0.59
11	16.61	17.55	5.05	5.01	0.3	0.29
12	12.79	14.47	1.61	13.89/1.89	0.13	0.94/0.13
13	12.73	12.70	7.64	5.87	0.6	0.46

n.d. = not developed.

All measurements in mm.

**Table 3:** Tooth height and the R/F ratio in the upper jaws of IVPP V12633.

Number of alveolus	Total height of functional tooth (F)		Total height of replacement tooth (R)		R/F ratio	
	left	right	left	right	left	right
1	6.77	6.50	—	4.04	—	0.62
2	8.33	7.56	—	n.d.	—	n.d.
3	9.30	9.02	—	n.d.	—	n.d.
4	9.70	9.66	—	5.35	—	0.55
5	10.70	10.96	—	2.59	—	0.24
6	10.38	9.93	—	9.90	—	0.99
7	11.34	10.13	—	4.03	—	0.4
8	9.89	10.06	—	n.d.	—	n.d.
9	8.62	8.80	—	4.74	—	0.54
10	6.73	6.72	—	n.d.	—	n.d.

n.d. = not developed.

All measurements in mm.

**Table 4:** Tooth height and the R/F ratio in the upper jaws of CAGS-IG-VD-002.

Number of alveolus	Total height of functional tooth (F)		Total height of replacement tooth (R)		R/F ratio	
	left	right	left	right	left	right
1	5.28	5.11	1.17	n.d.	0.23	n.d.
2	7.19	7.06	n.d.	n.d.	n.d.	n.d.
3	8.49	8.3	3.92	2.71	0.47	0.32
4	10.07	9.86	1.47	1.36	0.15	0.14
5	7.57	7.15	n.d.	n.d.	n.d.	n.d.
6	9.96	10.16	3.70	3.33	0.36	0.33
7	9.59	10.66	1.43	1.42	0.13	0.15
8	9.28	8.91	4.12	4.80	0.46	0.52
9	8.78	8.05	1.98	2.01	0.25	0.23
10	5.08	3.43	n.d.	n.d.	n.d.	n.d.

n.d. = not developed.

All measurements in mm.

**Table 5:** Tooth height and the R/F ratio in the lower jaw of IVPP V12738

Number of alveolus	Total height of functional tooth (F)		Total height of replacement tooth (R)		R/F ratio	
	left	right	left	right	left	right
1	8.99	8.15↑	2.59	5.25	0.29	0.64
2	9.08	8.31↑	n.d.	1.79	—	0.22
3	7.30↑	7.47	n.d.	1.96	—	0.26
4	6.81	7.67	n.d.	7.69	—	1.00
5	13.64	10.43	4.20	n.d.	0.31	—
6	12.52	13.62	1.47	8.01	0.12	0.59
7	15.64	12.23	8.11	3.15	0.52	0.26
8	11.64	17.32	3.64	13.77/1.29	0.31	0.80/0.07
9	17.35	10.66	13.28	6.28	0.77	0.59
10	17.71	17.39	6.81	3.96	0.38	0.23
11	12.41	15.81	2.82	11.28/0.90	0.23	0.71/0.06
12	15.16	15.63	9.18	5.44	0.61	0.35
13	10.43	14.45	3.88	10.20	0.37	0.71
14	10.75	6.45	9.03	3.99	0.84	0.62
15	5.65	10.35	2.73	n.d.	0.48	—

n.d. = not developed.

All measurements in mm.

**Table 6:** Tooth height and the R/F ratio in the lower jaw of IVPP V12633.

Number of alveolus	Total height of functional tooth (F)		Total height of replacement tooth (R)		R/F ratio	
	left	right	left	right	left	right
1	6.89	4.69	1.31	1.36	0.19	0.29
2	7.66	7.46	n.d.	n.d.	—	—
3	4.67	9.97	4.81	4.25	1.03	0.43
4	8.44	10.70	1.58	1.79	0.19	0.17
5	9.26	10.57	n.d.	n.d.	—	—
6	7.49	11.39	7.24	4.41	0.97	0.39
7	11.23	7.90	3.19	2.74	0.28	0.35
8	9.46	9.75	10.07	9.79	1.06	1.00
9	11.63	8.90	4.15	4.44	0.36	0.50
10	9.96	5.58	1.19	n.d.	0.12	—
11	8.26	6.87	4.12	3.23	0.50	0.47
12	5.40	5.97	n.d.	n.d.	—	—

n.d. = not developed.

All measurements in mm.

**Table 7:** Tooth height and the R/F ratio in the lower jaw of CAGS-IG-VD-002.

Number of alveolus	Total height of functional tooth (F)		Total height of replacement tooth (R)		R/F ratio	
	left	right	left	right	left	right
1	—	3.52↑	—	n.d.	—	—
2	—	3.92↑	—	1.98	—	0.51
3	—	6.30	—	1.09	—	0.17
4	—	8.33	—	n.d.	—	—
5	—	4.44	—	6.05	—	1.36
6	—	10.34	—	2.88	—	0.28
7	—	9.63	—	1.19	—	0.12
8	—	10.68	—	5.06	—	0.47
9	—	9.13	—	2.41	—	0.26
10	—	6.56	—	n.d.	—	—
11	—	6.10	—	1.89	—	0.31
12	—	2.96	—	n.d.	—	—

n.d. = not developed.

↑ = the true size should be higher for the crown was lost.

All measurements in mm.

## Discussion

### Symmetry in replacement

**Figure 5:**
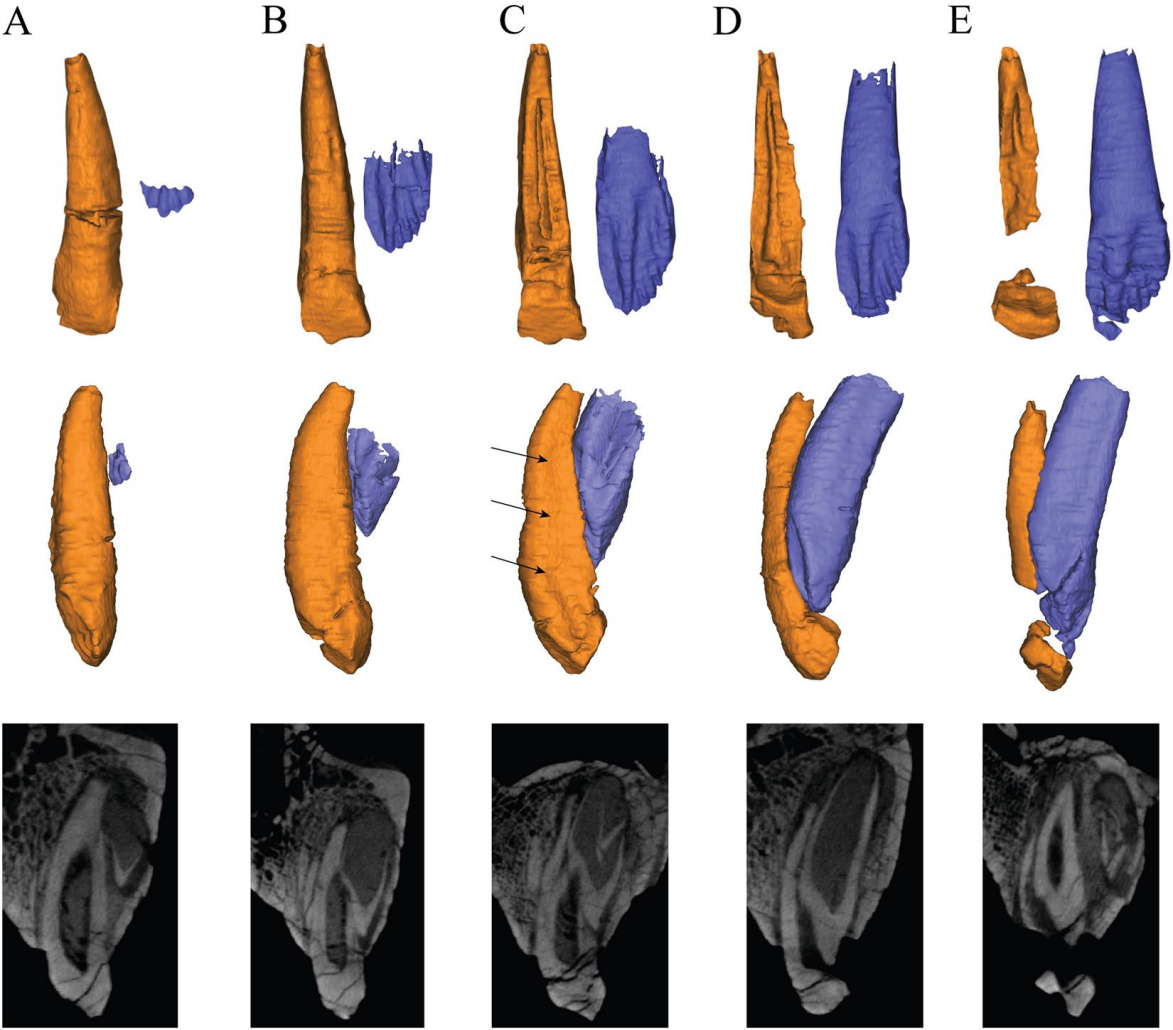
Schematic representation of the dentition in the four jaw quadrants of the three *Liaoceratops yanzigouensis* specimens. A. IVPP V12738. B. IVPP V12633. C. CAGS-IG-VD-002. In the upper quadrants, the premaxilla is separated from the maxilla by a line. Numbers indicate tooth positions from mesial to distal. open circles: the lost teeth; gray circles: replacement teeth; gradient color circles: broken teeth; functional teeth: black circles, small = size smaller than 5 mm, medium = between 5 mm to 10 mm, large = larger than 10 mm. The replacement teeth have been precisely located with regard to the functional teeth they will replace. The height of bars equal to the ratio of replacement tooth and their corresponding functional tooth. Black bars: ratios of the left; gray bars, ratios of the right.

To determine whether or not tooth replacement waves progressed symmetrically between different tooth bearing elements, we made pairwise comparisons of the dorsal-ventral heights of erupted teeth as well as of F/R ratios at each position in the maxillae and dentaries of each of the three specimens (Tables 1–7 and Fig. 5), following in part methods used on other extant^47–49^ and extinct^17,50^ reptiles. Prior studies generally only consider the ratio between replacement and functional teeth at each position, but we chose to also analyze symmetry of functional crown height, as the teeth of *Liaoceratops* show extensive wear along the entire tooth rows providing evidence for complex interaction between upper and lower dentitions during feeding. As a statistical test of whether replacement was symmetrical between two jaw elements, we used Kendall’s Tau to determine whether functional crown heights/ F/R ratios on one jaw were significantly correlated with the same positions in the other jaw, or not. This non-parametric test was selected because tooth size varies considerably along the length of the tooth row, and absolute and proportional differences may thus vary considerably for a given tooth position. Likewise, maxillary (M) and dentary (D) teeth differ from one another in size and shape^7^. By applying a test that uses ranked values we avoid biases that may arise from differences in tooth size. Because the holotype specimen has two more teeth in each dentary than in the maxillae, we compared the first ten tooth position in each as new tooth positions are typically added at the distal end of the tooth row in reptiles^43^. Analyses were conducted in the software PAST ver. 3.0^51^ and correlation coefficients and their p-values are provided in Table 8.

**Table 8:** Correlation coefficients (Kendall’s Tau) and associated p-values for ranked correlations between jaws in the three studied specimens of *Liaoceratops*.

SPECIMEN	TOOTH HEIGHT		R/F RATIO	
	TAU	P-VALUE	TAU	P-VALUE
IVPP V12738 M/M	0.7187	0.00017994	0.96364	3.69*10^−5^
IVPP V12633 M/M	0.8667	0.00048616	NA	NA
CAGS-IG-VD002 M/M	0.8222	0.00093503	0.6	0.0904
IVPP V12738 D/D	0.39048	0.042461	−0.15913	0.52185
IVPP V12633 D/D	0	1	0.6128	0.03221
IVPP V12738 M/D	0.61397/0.58066	0.025599/0.0057243	0.1972	0.45921
CAGS-IG-VD002 M/D	0.67404	0.032573	0.8000	0.050044
IVPP V12633 M/D	0.17945/0.55556	0.61985/0.025347	0	1

For CAGS-IG-VD002 and the R/F ratio for IVPP 12633, only the right maxilla and dentary were analyzed for M/D comparisons; other M/D results are listed as left/right.

The greatest degree as in these ratios (Table 2), (i.e. the most “symmetrical” tooth wave patterns) are observed between the upper left and right maxillae of the holotype (Fig. 5A; Table 8) as measured by both functional tooth height, and for the holotype, also for the R/F ratio. All of these have Tau values greater than 0.7 and highly significant relationships. There is no poor to no symmetry in replacement patterns between dentaries, and only poor to moderate symmetry between opposing maxillary and dentary toothrows. These results indicate that the degree of tooth wear and the complex oral processing patterns it provides evidence for are not predicated on synchronous patterns of tooth eruption between opposing toothrows.

Similar patterns of symmetry in replacement patterns have been reported in various reptiles previously, but not yet in dinosaurs. Cooper^52^ found that in the lizard *Lacerta*, bilateral symmetry was common in tooth replacement patterns. Cooper^47^ studied tooth replacement in the slowworm, *Anguis fragilis*, and bilateral symmetry was seen in tooth replacement patterns as well as some symmetry between patterns across the upper and lower jaws. Miller and Radnor^48^ examined the skulls of 15 young *Caiman sclerops* and found that bilateral symmetry was present between the maxillary toothrows of three specimens. Cong *et al*.^49^ examined two young *Alligator sinensis* specimens and found that bilateral symmetry in tooth replacement stages averages 81%. Similar patterns were also reported in the Sauropterygia, such as placodonts^50^, and in the rostral dentition of the pliosaurs *Pliosaurus kevani* and *Peloneustes*^17^. In *Chalcides* (Squamata; Scincidae)^53^, symmetrical replacement patterns are, however, absent according to Delgado *et al*., who concluded that synchronous replacement did not affect feeding functions in this taxon. Berkovitz^54^ and Sassoon *et al*.^17^ believed that symmetrical (i.e. synchronous between different jaws) tooth replacement would not diminish the effectiveness of hunting ability during the period of replacement process, for the overall integrity of the jaw is not compromised because adjacent functional teeth perform the required function. All of the cited studies were on carnivorous reptiles, however, and our study provides an insight on degrees of symmetry in tooth replacement in a herbivorous dinosaur exhibiting a substantial degree of tooth wear.

### Replacement process

**Figure 6:**
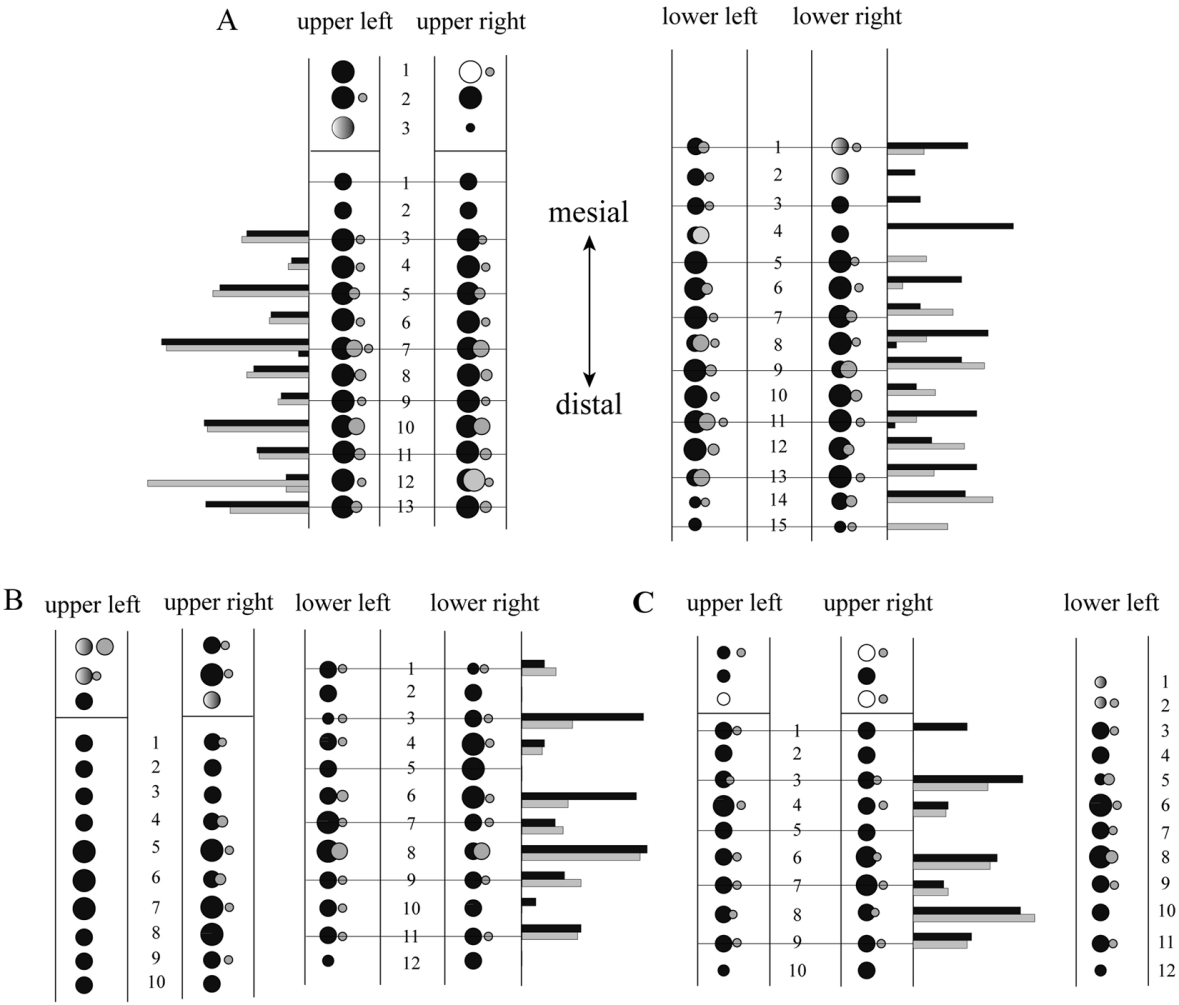
The tooth replacement process illustrated by teeth at different stages of eruption/replacement in the maxilla of the holotype. Elements in the CT reconstructions are colour-coded as follows: functional teeth, orange; replacement teeth, blue. Upper row, the functional teeth in lingual view, the corresponding replacement teeth are extracted separately to show their labial sides. Middle and lower rows, series of stages in the successional cycle seen in transverse sections in 3D construction and CT scans. Scale bars equal 5 mm. (**A**) A recently, fully erupted tooth crown (right tooth 4). (**B**) Growth of the replacement tooth and initiation of root resorption (right tooth 8). (**C**) Growth of successional tooth and the enlargement of the resorbed area on the functional tooth (right tooth 10). The arrows represent the the shallow grooves on mesial side. (**D**) Large part of functional root resorbed; tip of the successional tooth moving close to the functional tooth crown. In some tooth position, a second successional germ tooth makes its appearance (right tooth 7). (**E**) Functional crown about to be shed, root remnant present labial to replacement crown; first successional tooth about to erupt (right tooth 12).

With the exception of *Fruitadens*^55^, no 3D reconstruction has been published with the aim of analyzing the tooth replacement in a basal ornithichian or basal ceratopsian. In *Liaoceratops*, tooth replacement patterns are similar across the juvenile and adult specimens studied. During the replacement process, the crown of a replacement tooth appears adjacent to the lingual wall of functional tooth root. The newly formed tooth crown is situated lingual and a small distance away from the root of the functional tooth that it will replace (Fig. 6A). This pattern is similar to other dinosaurs including theropods and most other amniotes^8,55,56^ but differs slightly from the conditions in posthatchling crocodylians, mammals and early birds^42^, which exhibit subdental replacement. The replacement tooth grows at an angle to the long axis of the tooth it will replace. Its crown comes into contact with the lingual surface of the root of the functional tooth it is replacing (Fig. 6B) and begins to push into the dentine and even the pulp cavity of its predecessor. At this phase of eruption, the crown of a new tooth is fully developed but the root is short with a wide pulp cavity (Fig. 6C).

During the process of resorption, the labial surface of the functional tooth appears unaffected and still functional although the lingual surface has been heavily resorbed (Fig. 6D). In CT scan images (Fig. 2A,B), the heavily resorbed functional teeth were not ankylosed to the jaws and probably held together by periodontal ligament, as inferred for other dinosaurs. A tooth in an advanced stage of resorption is very weak, with the root reduced to a thin splint of dentine with an exposed pulp cavity. At some tooth positions (e.g. tooth 12 in the left maxilla of the holotype, Fig. 2D), a highly resorbed but functional tooth may still be present, while the replacement tooth is well developed and a new germ tooth is visible lingual to its root (Fig. 4C,E). In *Liaoceratops*, the functional crowns may still be present even when the replacement teeth had reached about 80% or even 90% of their predicted full-grown size. By contrast, in the pterosaur *Coloborhynchus robustus* (Pterosauria), functional crowns were shed when their replacement had reached about 60% of the full-grown height^16^, and in crocodiles, crowns are shed when replacement teeth reach about half their full-grown size^56^. Finally, the previous functional tooth would cast off and the cycle begins again. As proposed by Edmund^8^, which produce repeating wave-like patterns along the jaw.

### Tooth replacement pattern

In the holotype of *Liaoceratops*, there are two new erupted replacement teeth present in at least one alveolus (Fig. 2G) possibly presaging the potential to have more than one replacement tooth per tooth family, a derived feature of ceratopsid tooth batteries. In basal euornithopods, there are only two generations of teeth per tooth position^16^. However, *Liaoceratops* indicates that more complicated patterns of tooth replacement can evolve at relatively small body size in ornithischians.

**Figure 7:**
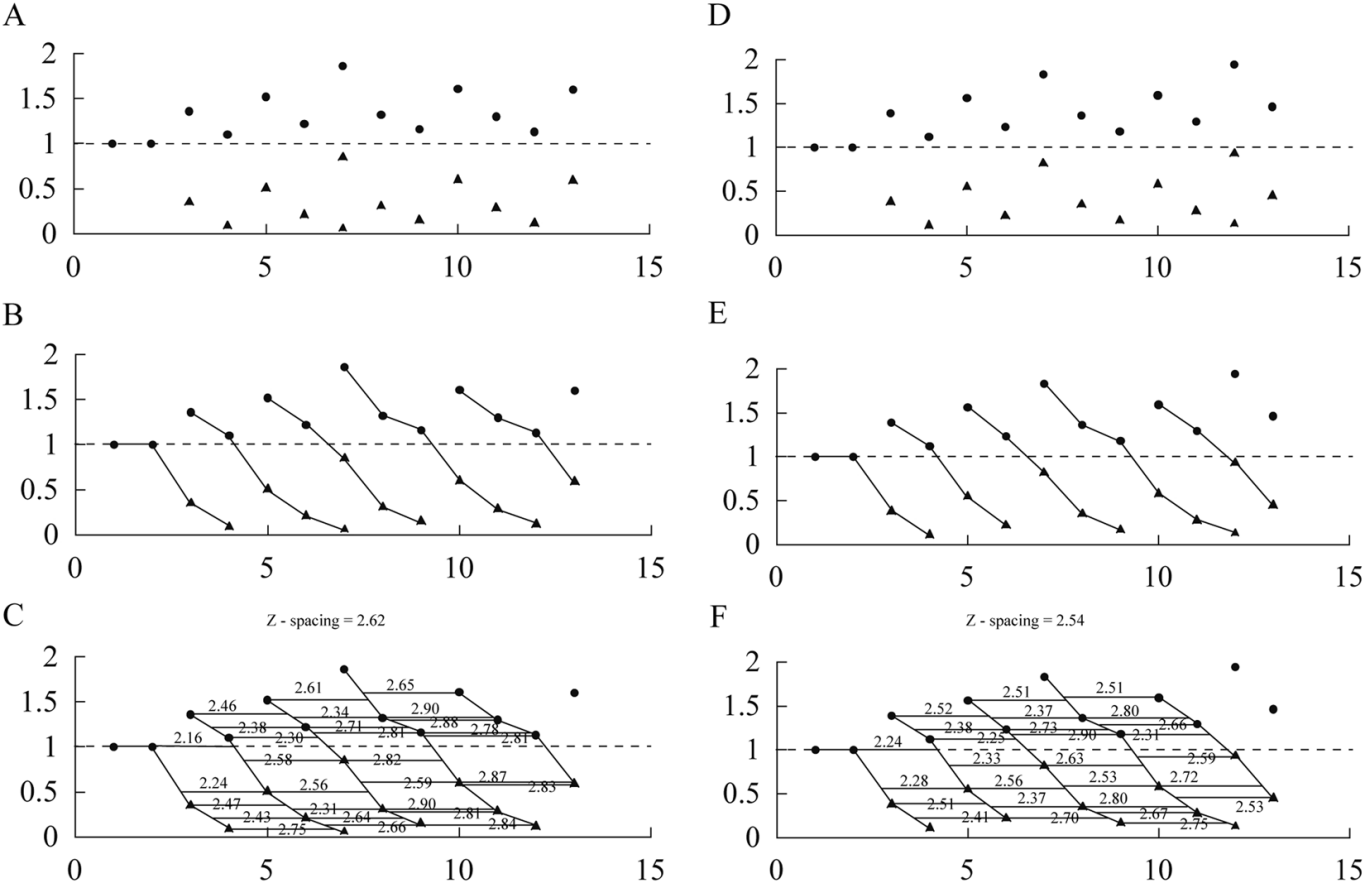
Quantification of upper jaw dentition in IVPP V12738. X-axis is the tooth position, Y-axis is the tooth replacement index. (**A–C**) are upper left side of jaw, (**D–F**) are upper right side of jaw. A and D: Replacement index. B and E: Zahnreihen. C and F: Z-Spacing. Dark circles representing the replacement index of functional teeth of upper jaw, triangle representing the replacement index of replacement teeth of upper jaw.

The general tooth replacement pattern in *Liaoceratops yanzigouensis* is just agrees with Edmund^8^, that the dental pattern in the basal ceratopsians apparently followed a course similar to that in the basal ornithopods. The replacement teeth are on the lingual side of the alveoli and are lingual to the functional teeth. The measurements of the teeth and the reconstructed dentition has been transfered to the replacement index shown in Fig. 7. From these data, the Zahnreihen were constructed. The result of the measurement is a Z-Spacing ranging from 2.16 to 2.90 with a mean value of 2.58 in the skull of the holotype (Fig. 7C,F). The value is therefore within the range of 1.56–2.8 reported by DeMar^21^ typical for reptiles. The spacing between Zahnreihen ranges from 2.2 to 2.3 in *Parksosaurus warreni* and *Hypsilophodon foxii*, to 3.0 in *Zephyrosaurus schaffi*^57^. The direction of the replacement waves could be deduced and the replacement waves were constructed based on the Z-Spacing. The waves obviously indicated that the replacement waves proceeded from back to front^51^, with replacement beginning at the anterior end of each wave and becoming complete at its posterior end.

There are only few reports on resorbed tooth remnants in the jaws of dinosaurs, and they have occasionally been observed in *Crocodilus niloticus*^56^ and are found in the adult gobiid fish, *Sicyopterus japonicus* and *Sicydium plumieri*^58–60^. Poole^56^ believed the retention of a functional tooth remnant in the socket is an anomalous condition in crocodiles caused by unusual circumstances. In the specimens of *Sicyopterus japonicus*, the old functional teeth are not shed, but resorbed and subsequently degraded^50^.

By contrast, our CT sections and 3D reconstructions reveal that remnants of mostly resorbed functional teeth are present in both the upper and lower dentition of *Liaoceratops* and in both juvenile and adult specimens, arguing against their being anomalies in this taxon. The stages of tooth replacement and eruption described and illustrated in Fig. 7 provide insights into why these remnants are so prevalent. Unlike *Psittacosaurs*, *Pachycephalosaurs*, and basal neornithischians, which all have conical tooth roots with a subcircular cross section, *Liaoceratops* and other basal neornithischians have maxillary and dentary teeth with roots that are elliptical in cross section and wider labiolingually than mediodistally. Furthermore, neoceratopsian teeth including those of *Liaoceratops* are curved along their long axis so that the labial face of maxillary tooth is strongly convex apicobasally. As a replacement tooth resorbs the root of a functional tooth lingually and migrates up the alveolus, the far labial side of the root lies beyond the zone of resorption and thus is retained as a remnant hidden in the jaw. This process is not only a function of the derived anatomy and curvature of the *Liaoceratops* dentition, but is also due to the angle at which the replacement tooth forms and the relatively crownward point at which it contacts and begins to resorb the root of the functional tooth. Lingual formation of germ teeth and initiation of resorption from the lingual side of functional teeth is the ancestral state for archosaurs^8^, but until recently there was little published evidence for root remnants of replaced teeth, suggesting that replacement involved complete resorption of the preceding teeth. Recent micro CT^55^ or histological e.g.^42,43,61,62^ examination of reptile jaws and dentitions has revealed that tooth replacement does not always lead to complete resorption, however, with remnants of tooth roots or alveolar bone sometime buried in interdental bone. However, *Liaoceratops* represents the first amniote for which multiple generations of tooth remnants (i.e. traces of tooth families) are documented. As demonstrated by the increasing size of successive root remnants in the tooth families of the holotype specimen of *Liaoceratops* (Fig. 4), successive generations of replacement teeth were larger than the functional teeth they replaced in *Liaoceratops*, even though replacement did not result in full resorption of the preceding functional tooth roots. One ready explanation for this phenomenon may be that the jaw bones grew progressively wider at a greater rate than in other examined clades, resulting in a relative lingual migration of the dental lamina over development. Indeed, the dentary of *Liaoceratops* is relatively massive as noted by Xu *et al*.^7^ and the maxilla is also very broad in occlusal view, especially at its caudal end (Fig. 1D), when compared to *Psittacosaurus* and other marginocephalians. Determination of whether remnants of replaced teeth occur in other basal neoceratopsians, will require more extensive sampling using CT or other radiographic methods. Given the similarities in anatomy and replacement patterns between *Liaoceratops, Archaeoceratops*, and *Auroraceratops* described by Tanoue *et al*.^4^ information on the latter two taxa would be very useful to establishing how widespread this phenomenon may be.

Functional teeth are replaced by progressively larger teeth in order to keep up with the growing jaws. Presumably then, size differences between tooth generations provide an idea of how much time passes between formation of successive teeth. The evidence of extensive tooth migration between replacement events may be helpful in determining how much time has passed between replacement cycles. Interestingly in captorhinids, a basal clade of eureptiles that have multiple rows of marginal teeth, the old function teeth would be shed and the remains would still remain labial to the new tooth^60^.

One of the key characters of ceratopsids, in which they differ from basal ceratopsians, is the evolution of the dental battery. The dental battery in ceratopsids is superficially similar to that of the iguanodontians and hadrosaurids^8^ in that the dental battery of ceratopsids holds three to five teeth in each alveolus, with adjacent tooth crowns tightly packed against one another^2^. Ceratopsid teeth are distinguished from those of hadrosaurids, however, by the presence of subdental replacement of deeply roots that are heavily resorbed by replacement tooth in the series so that they become bifid in all but the youngest germ tooth^42^.

Shallow longitudinal grooves on the roots of maxillary and dentary teeth are reported in more derived basal ceratopsian dinosaurs such as *Protoceratops*^63^ and *Zuniceratops*^64^, and may represent an initial step in the evolution of ceratopsid bifid roots, as they became deeper and eventually split a root into two prongs^9^. In basal neoceratopsians, these grooves serve to accomodate adjacent crowns in neighboring tooth families allowing for closer packing of the dentition, another incipient stage in the evolution of the complex ceratopsid dental battery. Faint mesial grooves are present on the crownward part of the root of at least some teeth of *Liaoceratops*, including tooth position 10 (Fig. 5C) in the right maxilla of the holotype. This groove does not appear to be created by partial root resorption from neighboring teeth like hadrosaurid teeth^62^ for the teeth are not tightly packed. Together with the occasional presence of two generations of replacement teeth in at least the holotype of *Liaoceratops*, early stages of two important structural traits required for the evolution of ceratopsid tooth batteries make their phylogenetically earliest (if subtlest) manifestation of this trait in this keystone taxon. Furthermore, the presence of a dead functional crown that was completely separated from its root by a replacement crown at tooth position 12 in the holotype may presage the ceratopsid condition in which the entire erupted row of functional crowns is dead as their roots are resorbed^42^.

## Methods

### Computed Tomography

We use high resolution X-ray micro-computed tomography to reveal internal anatomical features of tooth replacement in the maxilla and dentary. Scanning was carried out using a customized 225 kV micro-computed tomography instrument (developed by the Institute of High Energy Physics, Chinese Academy of Sciences (CAS)) at the Key Laboratory of Vertebrate Evolution and Human Origins, CAS. All three specimens were scanned at a beam energy of 140 kV and a flux of 100uA with a detector resolution of 42.3 um per pixel while using a 360° rotation with a step size of 0.5° and an unfiltered aluminium reflection target. A total of 720 transmission images were reconstructed in a 2,048*2,048 matrix of 1,536 slices using a dedicated two-dimensional reconstruction software developed by the Institute of High Energy Physics, CAS. Scanning was also carried out using a 450 kV micro-computed tomography instrument to scan the skull of the holotype of *Liaoceratops*, IVPP V12738, for an overall view. The specimen was scanned with beam energy of 430 kV and a flux of 1.5 mA at a detector resolution of 160 um per pixel using a 360° rotation with a step size of 0.25° and an unfiltered aluminium reflection target. A total of 1,440 transmission images were reconstructed in a 2,048*2,048 matrix of 2,048 slices.

### Three-Dimensional Modeling and Meshing

3D models were rendered from µCT data for study in the tooth replacement. Digital modeling and processing was made with the 3D analysis software Mimics® (Materialise Corporation, Leuven, Belgium, versions 15.0 and 16.0). The program builds meshes for based on density differences in each specimen, and apply material properties to each mesh. The skull of IVPP V12738 was scanned in both 450 kV and 225 kV micro-computerized tomography. Both CT datasets were input in Mimics to render 3D models. The former model (450 kV CT) is for an overall view of the skull and the latter (225 kV CT) is for the close-up detail of tooth replacement.
